# Understanding Diabetes and Obesity in The Bahamas Through International Comparison of Health, Economic, and Policy Indicators

**DOI:** 10.7759/cureus.91036

**Published:** 2025-08-26

**Authors:** Cesar Barrabi, Camren Adams, Omur C Elci

**Affiliations:** 1 Epidemiology and Public Health, The Chicago School, Chicago, USA; 2 Medical Education, Western Atlantic University School of Medicine, Freeport, BHS

**Keywords:** caribbean health, diabetes, gender disparities, health disparities, non-communicable diseases, obesity, public health policy, socioeconomic determinants, the bahamas, undiagnosed diabetes

## Abstract

Background

Diabetes mellitus is a global issue affecting over 828 million people in 2021. Risk factors for developing diabetes include poor diet, sedentary lifestyle, and genetic predispositions; however, growing evidence suggests a significant influence from socioeconomic determinants. The Non-Latin Caribbean continues to be an underrepresented population in diabetes research, particularly in The Bahamas. In this paper, we investigate the current state of diabetes in The Bahamas and examine the socioeconomic determinants associated with poor health outcomes. This study aimed to evaluate diabetes prevalence, premature mortality, underdiagnosis, obesity trends by sex, food insecurity, inequality, and obesity-related policy implementation in The Bahamas compared with non-Latin Caribbean and high-income countries, to identify national patterns of disease burden and inform targeted public health policy.

Methods

Publicly available data were compiled from the World Health Organization, Food and Agriculture Organization, International Diabetes Federation, Pan American Health Organization (PAHO) Enlace, World Bank, and regional reports, prioritizing estimates from 2019 to 2022. With a focus on The Bahamas, we collected data from 10 non-Latin Caribbean nations, as well as the United States, Canada, France, and Germany, to compare health and socioeconomic indicators and assess the current state of the Bahamas. Key metrics of interest included the prevalence of diabetes and obesity, diabetes-related mortality, and indicators of socioeconomic conditions.

Results

Diabetes prevalence in The Bahamas was lower than in the United States, but still fell within the higher range seen across the Caribbean. Premature mortality due to diabetes was considerably higher in The Bahamas compared to high-income countries and ranked among the highest in the region. When examining obesity trends, Bahamian women consistently demonstrated the greatest burden relative to both regional neighbors and high-income comparison countries. Across multiple socioeconomic indicators, The Bahamas also showed notable vulnerabilities and lagged behind North America in terms of policy response and implementation.

Conclusion

Diabetes in The Bahamas remains a serious public health concern, marked by high premature mortality and rising obesity rates that exceed those of several high-income countries. Strengthening national surveillance and addressing socioeconomic disparities will be critical for reversing current trends and supporting effective public health responses.

## Introduction

Diabetes mellitus is a growing public health crisis, with global prevalence rising sharply. By 2022, an estimated 828 million adults had diabetes, a dramatic increase since 1990 [1]. The Americas are particularly affected, with projections showing one in 11 adults in Latin America and the Caribbean will have diabetes by 2045 [2]. Despite increasing concern over diabetes and obesity in the Caribbean, comprehensive regional research remains limited, especially studies that examine how social conditions and healthcare access influence disparities in health outcomes.

Diabetes prevalence in the Caribbean has been linked not only to poor nutrition and physical inactivity but also to broader factors such as poverty, food insecurity, and limited access to healthcare [3]. Additionally, lower socioeconomic status, unemployment, and limited education are strongly linked to higher diabetes prevalence, especially among women [4,5]. Cultural and socioeconomic influences, rather than biological differences, likely explain why Caribbean women have higher obesity and diabetes rates than men. Healthcare access is inconsistent, with many nations lacking standardized screening, leading to high rates of undiagnosed diabetes [6,7]. Yet in the Caribbean, the broader social drivers of diabetes have received limited sustained attention, and policy responses remain fragmented despite the scale of the crisis.

Recent studies across the Caribbean have documented rising rates of diabetes and obesity, shaped by many of the same social and economic factors [8]. Multiple reviews have shown that women in the Caribbean are disproportionately affected by obesity, inactivity, and diabetes, while men tend to have higher rates of smoking and poor diet quality [3,9]. Socioeconomic and structural inequities shape these patterns, yet most countries lack recent data to guide interventions [10]. Regional studies also highlight the rising mortality burden of diabetes and its complications, calling for improved screening and targeted prevention efforts [11,12].

Obesity and diabetes remain pressing public health issues in The Bahamas, with earlier studies reporting disproportionately high rates among adolescents and women of lower socioeconomic status [13,14]. In response, national initiatives have promoted multi-sectoral approaches to obesity prevention, achieving some progress despite persistent policy and implementation gaps [15]. At the regional level, rising rates of type 2 diabetes have been attributed to increasingly sedentary lifestyles and widespread dietary changes, while local evidence points to a strong connection between food insecurity and chronic disease in Bahamian communities [16]. Yet despite this growing body of research, there remains a lack of up-to-date, comparative data on obesity and diabetes trends in The Bahamas, particularly in relation to high-income countries. This gap is especially important given emerging concerns about the appropriateness of standard diagnostic tools for use in Caribbean populations, which may obscure the true extent of disease burden [17].

This study aimed to evaluate diabetes and obesity trends in the Bahamas through international comparison with non-Latin Caribbean and high-income countries. Using publicly available data from 2019 to 2022, we examined diabetes prevalence, premature mortality, underdiagnosis, obesity trends by sex, food insecurity, inequality, and obesity-related policy implementation. The objective was to identify national patterns of disease burden, detection gaps, and structural vulnerabilities that may contribute to poor chronic disease outcomes in The Bahamas and to inform targeted public health policy.

This article was previously posted as a preprint on the medRxiv server on April 13, 2025 [31]. 

## Materials and methods

Data collection

Although geographically located in the Atlantic Ocean, The Bahamas is often included in Caribbean regional analyses due to its membership in the Caribbean Community (CARICOM) and its shared historical, cultural, and socioeconomic ties with Caribbean nations. Caribbean countries are generally composed of small island developing states with predominantly Afro-Caribbean populations and histories shaped by colonization, slavery, and migration. Despite geographic differences, the Bahamas faces similar health, economic, and policy challenges as its Caribbean neighbors. This study centers on The Bahamas (BHS) and includes comparisons with other sovereign Caribbean nations, the United States (USA), Canada (CAN), France (FRA), and Germany (DEU) as high-income reference points. Countries were selected based on the availability and completeness of data across key health and socioeconomic indicators. In addition to BHS, other non-Latin Caribbean nations such as Barbados (BRB), Belize (BLZ), Dominica (DMA), Grenada (GRD), Guyana (GUY), Saint Kitts and Nevis (KNA), Saint Lucia (LCA), Saint Vincent and the Grenadines (VCT), Suriname (SUR), and Antigua and Barbuda (ATG) were chosen to be included.

This study utilized publicly available data from the World Health Organization (WHO), International Diabetes Federation (IDF), World Bank, Pan American Health Organization (PAHO), Enlace, Caribbean Development Bank (CDB), Food and Agriculture Organization (FAO), International Monetary Fund (IMF), the WHO European Mortality Database, and national government reports (Tables 1-4). These sources compile standardized epidemiological, economic, and healthcare data from government health ministries, national surveys, hospital records, and economic reporting systems [18-22]. 

All indicators were retrieved between January and March 2025. For diabetes prevalence, mortality, and underdiagnosis, we primarily used IDF estimates and supplemented with PAHO ENLACE data. Obesity prevalence by sex was obtained from the WHO, while Human Development Index (HDI), Inequality-adjusted Human Development Index (IHDI), and Gini coefficient (GINI) were taken from UNDP and World Bank reports. Food insecurity and import dependency were sourced from FAO, and broader economic metrics came from CDB, IMF, and WHO. BHS was the focal point of analysis, and indicators were included only when data were available for BHS. Comparator countries were incorporated when at least two others in the region had data for the same period, balancing coverage with conciseness. When multiple estimates existed, the most recent standardized source was selected to ensure consistency. Analyses were descriptive and comparative in design.

To assess how countries are responding to obesity and diabetes risk at the policy level, we reviewed implementation data from the PAHO ENLACE regional platform. Twelve policy indicators were included, drawn from PAHO’s Plan of Action for the Prevention of Obesity in Children and Adolescents 2014-2019. These indicators cover areas such as fiscal policy, school nutrition, primary care, and food environment regulations. The specific measures assessed were (3.1.1) taxes on sugar-sweetened beverages; (3.2.1) restrictions on junk food marketing to children; (3.3.1) front-of-package nutrition labeling; (2.1.1) national school feeding programs; (2.1.2) limits on ultra-processed food sales; (4.1.1) a national obesity strategy; (4.2.1) open streets and physical activity initiatives; (4.3.1) support for family farming; (4.3.2) improved access and pricing for healthy foods; (1.1.1) obesity prevention through diet and activity in primary care; (1.2.1) publication of Code monitoring results; and (5.1.1) surveillance of obesity in women, children, and adolescents. Countries were scored as either “achieved” or “not achieved” based on national policy documents and regional reports compiled by PAHO.

Data analysis

All data processing and visualization (including scatterplots and trend lines) were conducted in Microsoft Excel (Redmond, USA). This dataset is a collection of country-level aggregates, not sampled data, meaning that within-group variability could not be assessed.

Data on diabetes prevalence and mortality from IDF were used to create a regional Geographic Information System (GIS) map of the Caribbean, generated in QGIS 3.34 T using GADM shapefiles performed by Fiver professional Ayesha Suraweera.

HDI and GINI comparisons were limited to The Bahamas and a small group of high-income countries selected for relevance and data reliability. These indicators are difficult to obtain across the wider Caribbean, and given this study’s focus on The Bahamas, comparisons were restricted to nations that provide meaningful context for interpreting national disparities.

Patient and public involvement

No patients or members of the public were involved in the design, conduct, analysis, or dissemination of this research.

## Results

High diabetes mortality persists despite regional variations in prevalence

After the data were collected, we began by examining diabetes prevalence and mortality rates across the Caribbean, based on 2021 data from IDF. Geographic differences were visualized using GIS mapping, with countries shaded according to their diabetes metrics, darker shades indicating higher values. This approach provides a spatial comparison of diabetes prevalence and mortality across the region, highlighting areas most affected by the disease.

**Figure 1:**
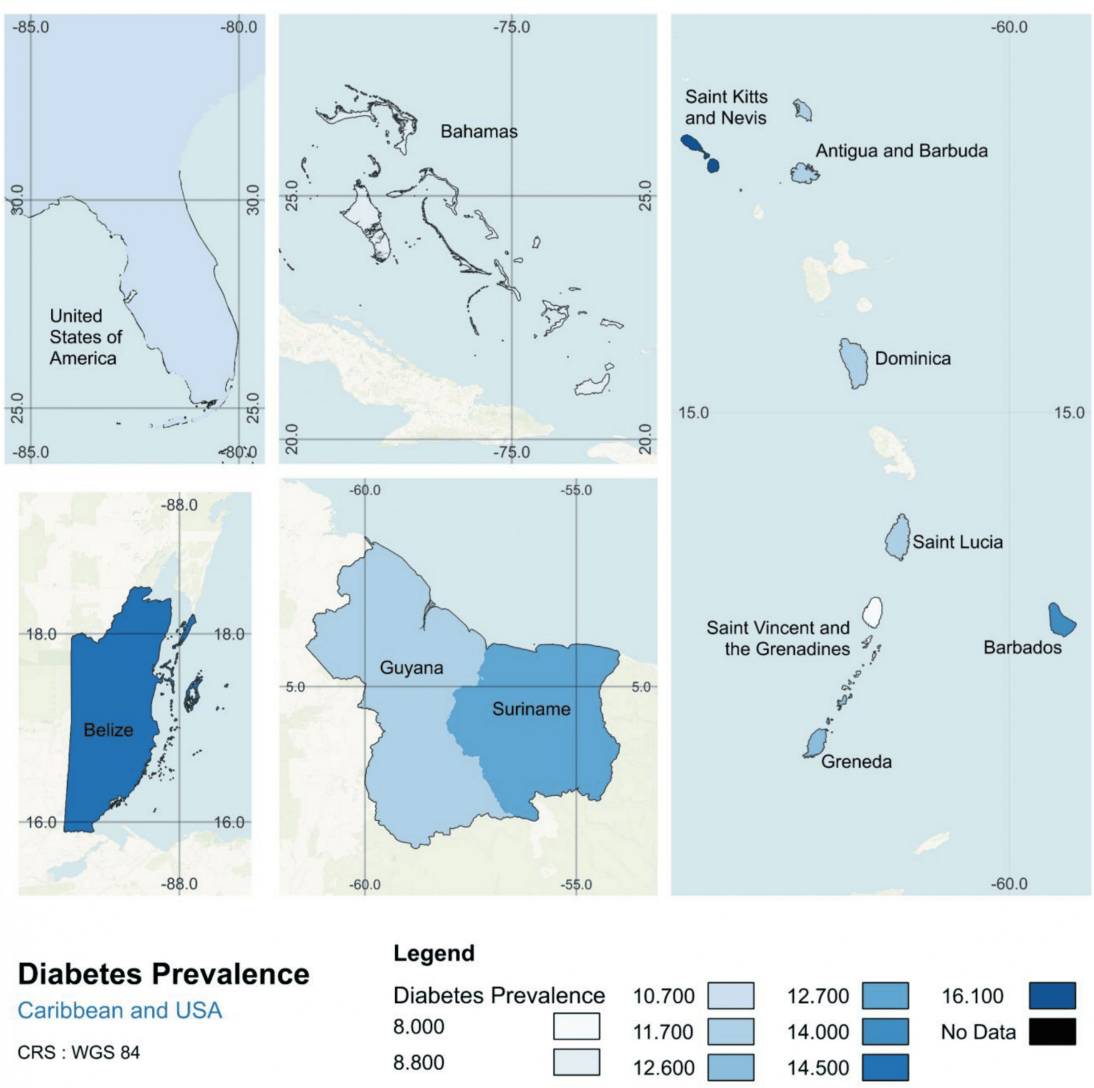
Regional patterns of diabetes prevalence in the non-Latin Caribbean and The Bahamas, 2021 All data were obtained from the International Diabetes Federation (IDF) by searching for diabetes prevalence indicators in 2021. The dataset includes estimates from national health surveys, surveillance programs, and modeled data where direct estimates are unavailable. IDF compiles data from peer-reviewed studies, national reports, and health ministries to generate country-level prevalence estimates. The map was created in QGIS 3.34 T using shapefiles from Global Administrative Areas (GADM) to visualize diabetes prevalence across Caribbean nations and the southeastern United States. Darker shades represent higher prevalence, highlighting regional variations in diabetes burden.

The results revealed significant variation in diabetes prevalence (Figure 1). The highest prevalence was observed in Saint Kitts and Nevis (16.1%), followed by Belize (14.5%) and Barbados (14.0%), while the lowest rates were reported in Saint Vincent and the Grenadines (8.0%), the Bahamas (8.8%), and the United States (10.7%). To provide a broader context, additional comparisons were made with France (5.3%), Germany (6.9%), and Canada (7.7%), which reported lower diabetes prevalence than most Caribbean nations.

**Figure 2:**
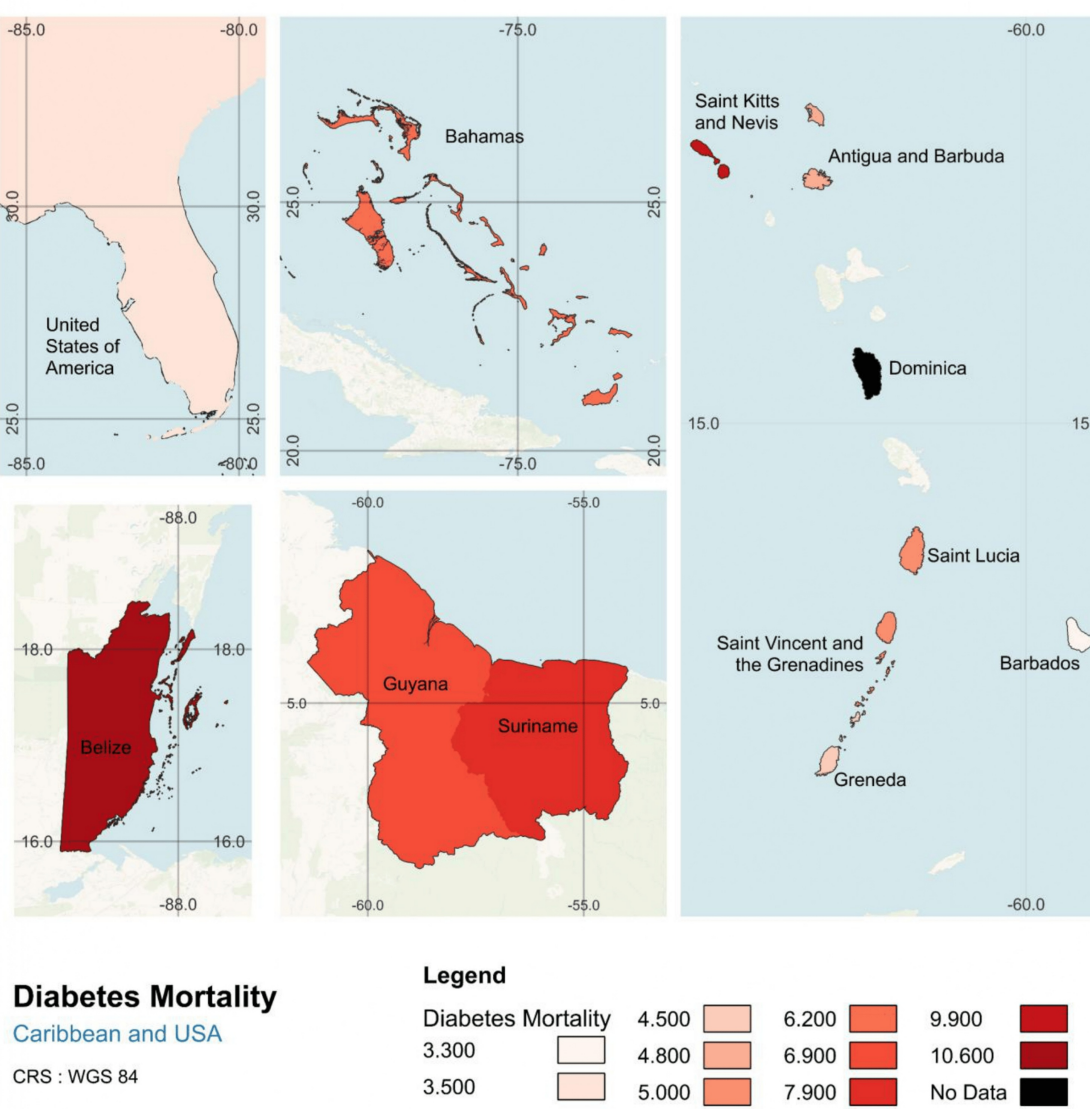
Diabetes-related deaths are disproportionately high in the non-Latin Caribbean and The Bahamas, 2021 All data were obtained from the International Diabetes Federation (IDF) by searching for diabetes mortality indicators in 2021. The dataset includes estimates from national health records, mortality databases, and modeled projections where direct data is unavailable. IDF compiles data from peer-reviewed studies, national reports, and health ministries to generate country-level mortality estimates. The map was created in QGIS 3.34 T using shapefiles from GADM to visualize diabetes-related mortality across Caribbean nations and the southeastern United States. Darker shades indicate higher mortality rates, highlighting disparities in diabetes-related deaths across the region.

In addition to prevalence, we also explored diabetes-related mortality, measured as the proportion of deaths occurring in individuals under 60 years (Figure 2). The highest mortality rates were observed in Belize (10.6%), Saint Kitts and Nevis (9.9%), and Suriname (7.9%), with the lowest rates recorded in the United States (3.5%), Barbados (3.3%), and Antigua and Barbuda (4.8%). The Bahamas ranked fifth in diabetes-related mortality among individuals under 60, with a rate of 6.2%, higher than the United States (3.5%), France (1.5%), Germany (1.7%), and Canada (2.0%), illustrating a striking difference in mortality rates.

The Bahamas presents a notable case, with a relatively low diabetes prevalence but the fifth-highest mortality rate (6.2%) among countries studied. According to 2021 IDF estimates, 28.9% of people with diabetes in The Bahamas were undiagnosed, more than double the proportion in the United States (12.5%), suggesting significant underdiagnosis.

Obesity and diabetes mortality trends in The Bahamas highlight severe gender disparities and regional divergence

**Figure 3:**
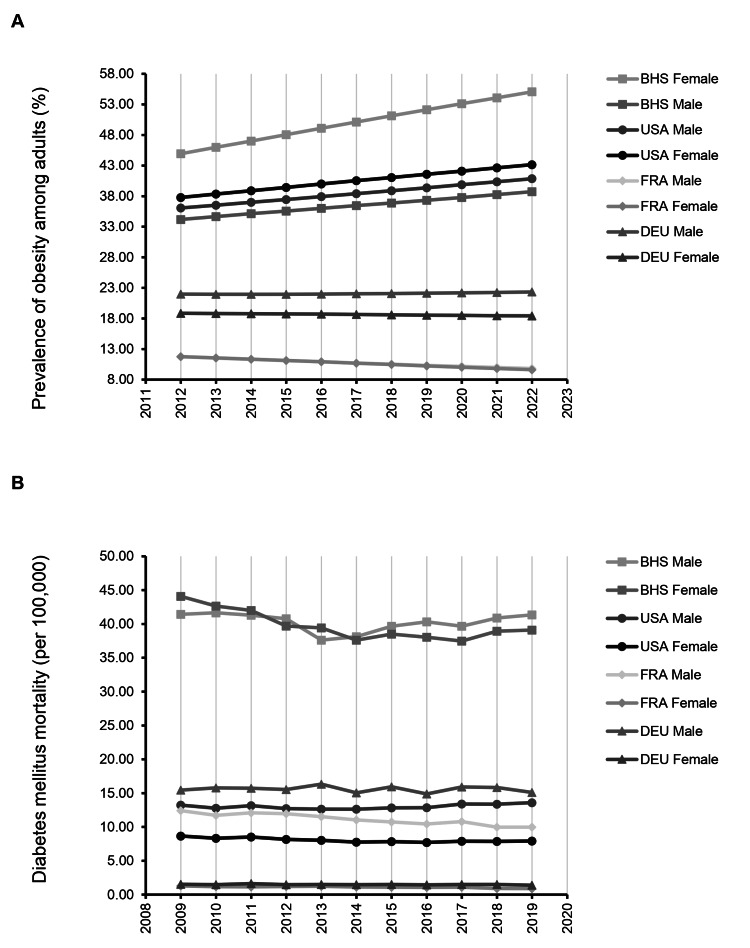
Rising obesity and persistent diabetes mortality reveal a gendered health crisis in the Bahamas All data are drawn from the Pan American Health Organization (PAHO) via the ENLACE regional health observatory. BHS: The Bahamas, FRA: France, DEU: Germany. (A) Line graph showing the prevalence of adult obesity (BMI ≥ 30 kg/m², age-standardized) by sex in The Bahamas from 2012 to 2022. Female obesity increased from 44.9% to 55.1%, the highest among all comparison groups. (B) Line graph showing age-standardized diabetes mortality per 100,000 by sex from 2009 to 2019. Mortality remained over five times higher than in the United States for women and more than triple for men. The combination of rising obesity and sustained mortality highlights serious gaps in prevention and care, with women experiencing the most severe outcomes.

To evaluate obesity and diabetes mortality in The Bahamas, we analyzed age-standardized data across the last decade and compared these findings to France (FRA), Germany (DEU), and the United States (USA). Our results reveal that Bahamian women face disproportionately high obesity levels, with rates increasing steadily from 44.9% in 2012 to 55.1% in 2022 (Figure 3A). In 2022, Bahamian women had the highest obesity prevalence across all groups, surpassing women in the United States (43.2%), Germany (18.4%), and France (9.6%). Obesity among Bahamian men also rose steadily from 34.2% in 2012 to 38.7% in 2022, but remained below male rates in the USA (40.9%) and above both Germany (22.3%) and France (9.8%).

Parallel patterns were observed for diabetes-related mortality (Figure 3B). From 2009 to 2019, The Bahamas consistently had the highest age-adjusted diabetes mortality across all groups. Among women, mortality ranged from 37.5 to 44.1 per 100,000, remaining nearly five times higher than rates observed in the United States (7.9 per 100,000) and over 40 times higher than in France (0.9 per 100,000) by 2019. Similarly, Bahamian men experienced persistently elevated mortality, with 2019 rates reaching 41.4 per 100,000, more than triple the U.S. rate (13.6) and well above Germany (15.1) and France (10.0). Despite having a lower diabetes prevalence than several other Caribbean nations, The Bahamas ranked fifth in diabetes mortality, suggesting substantial underdiagnosis and healthcare barriers. IDF data from 2021 further support this interpretation, with an estimated 28.9% of people living with diabetes in the Bahamas remaining undiagnosed, more than double the proportion in the United States (12.5%).

Food insecurity, import dependency, and inequality reveal structural vulnerabilities in The Bahamas

**Figure 4:**
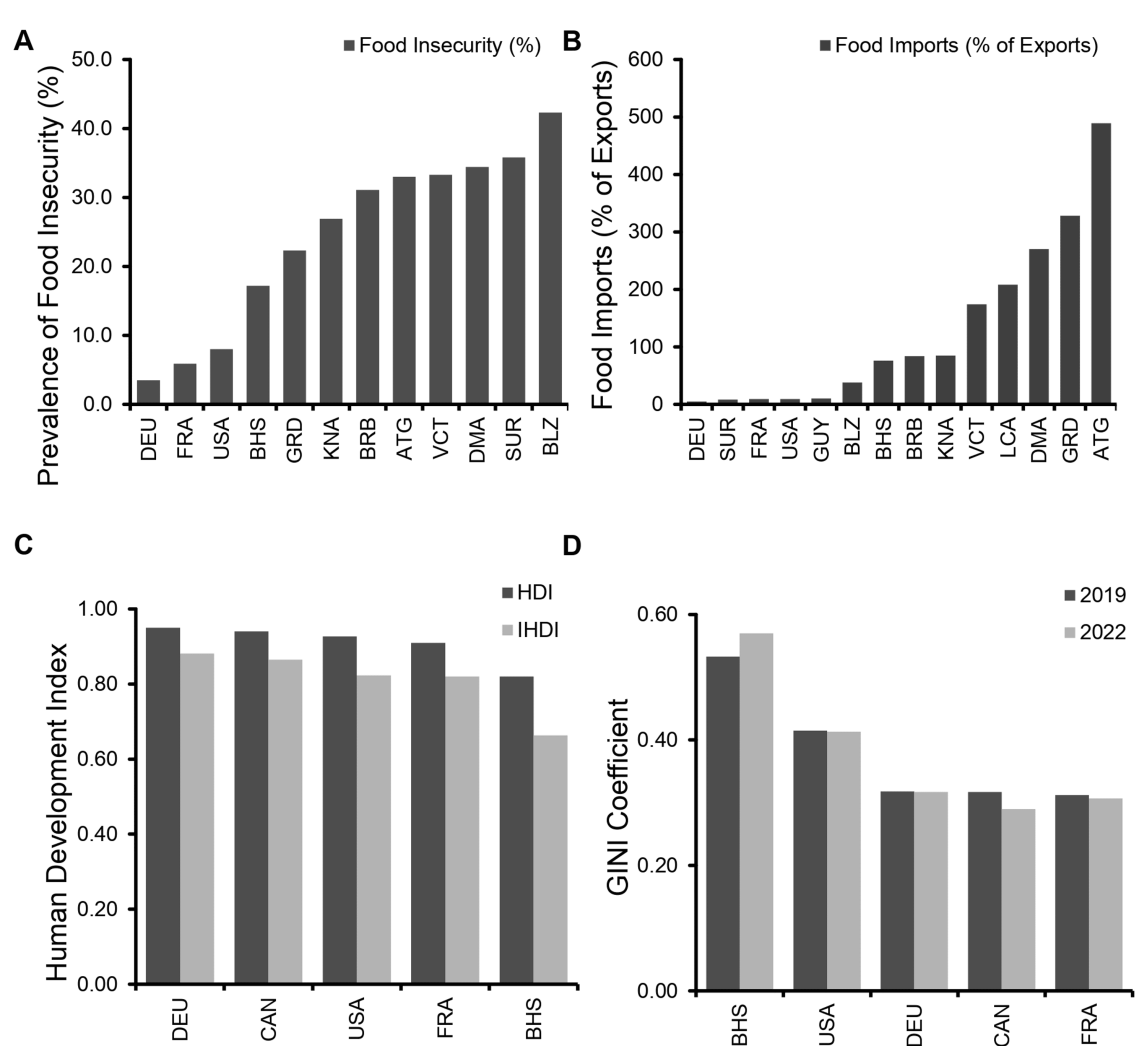
Socioeconomic conditions and inequality in the Bahamas compared to other nations Data were collected from the Food and Agriculture Organization (FAO) and the United Nations Development Programme (UNDP) to compare food insecurity, food import dependence, development indices, and income inequality across select Caribbean and high-income countries. ATG: Antigua and Barbuda, BHS: The Bahamas, BLZ: Belize, BRB: Barbados, CAN: Canada, DMA: Dominica, DEU: Germany, FRA: France, GRD: Grenada, GUY: Guyana, KNA: Saint Kitts and Nevis, LCA: Saint Lucia, SUR: Suriname, USA: United States, VCT: Saint Vincent and the Grenadines. (A) Food insecurity rates in 2022, with The Bahamas showing higher prevalence than France, Germany, and Canada, but lower than many Caribbean countries. (B) Food imports as a percentage of total exports, indicating high import dependency in The Bahamas. (C) Human Development Index (HDI) and inequality-adjusted HDI (IHDI) in 2022, reflecting a decline in overall development when accounting for inequality. (D) GINI coefficient trends from 2019 to 2022, showing increasing income inequality in The Bahamas relative to other countries.

To contextualize the social and economic factors influencing diabetes and obesity, we compared food security, economic dependency, and inequality across 12 Caribbean nations and four high-income countries (Figure 4). In 2022, food insecurity in the Bahamas was estimated at 17.2%, more than double the rate in France (5.9%), Germany (3.5%), and Canada (9.3%), but notably lower than many Caribbean neighbors such as Belize (42.3%) and Suriname (35.8%) (Figure 4A). Despite a relatively lower prevalence, The Bahamas remains heavily reliant on external food sources. Food imports accounted for 76% of total exports, reflecting a high level of dependency compared to countries like the USA (9%), France (9%), and Canada (2%) (Figure 4B).

National development indicators revealed further structural concerns. The Bahamas had a 2022 Human Development Index (HDI) of 0.82, comparable to France (0.91) and the USA (0.93), but its inequality-adjusted HDI (IHDI) dropped to 0.663, reflecting one of the largest development penalties among countries studied (Figure 4C). Similarly, inequality increased over time. The Bahamas had the highest GINI coefficient in the analysis, rising from 0.533 in 2019 to 0.57 in 2022, well above Germany (0.317), the USA (0.413), and Canada (0.29) (Figure 4D). These findings suggest that broad development gains have not translated into equitable outcomes. Instead, rising inequality, economic dependency, and food insecurity may be reinforcing health disparities, particularly in chronic disease risk.

Obesity policy progress lags in the non-Latin Caribbean compared to North America

**Figure 5:**
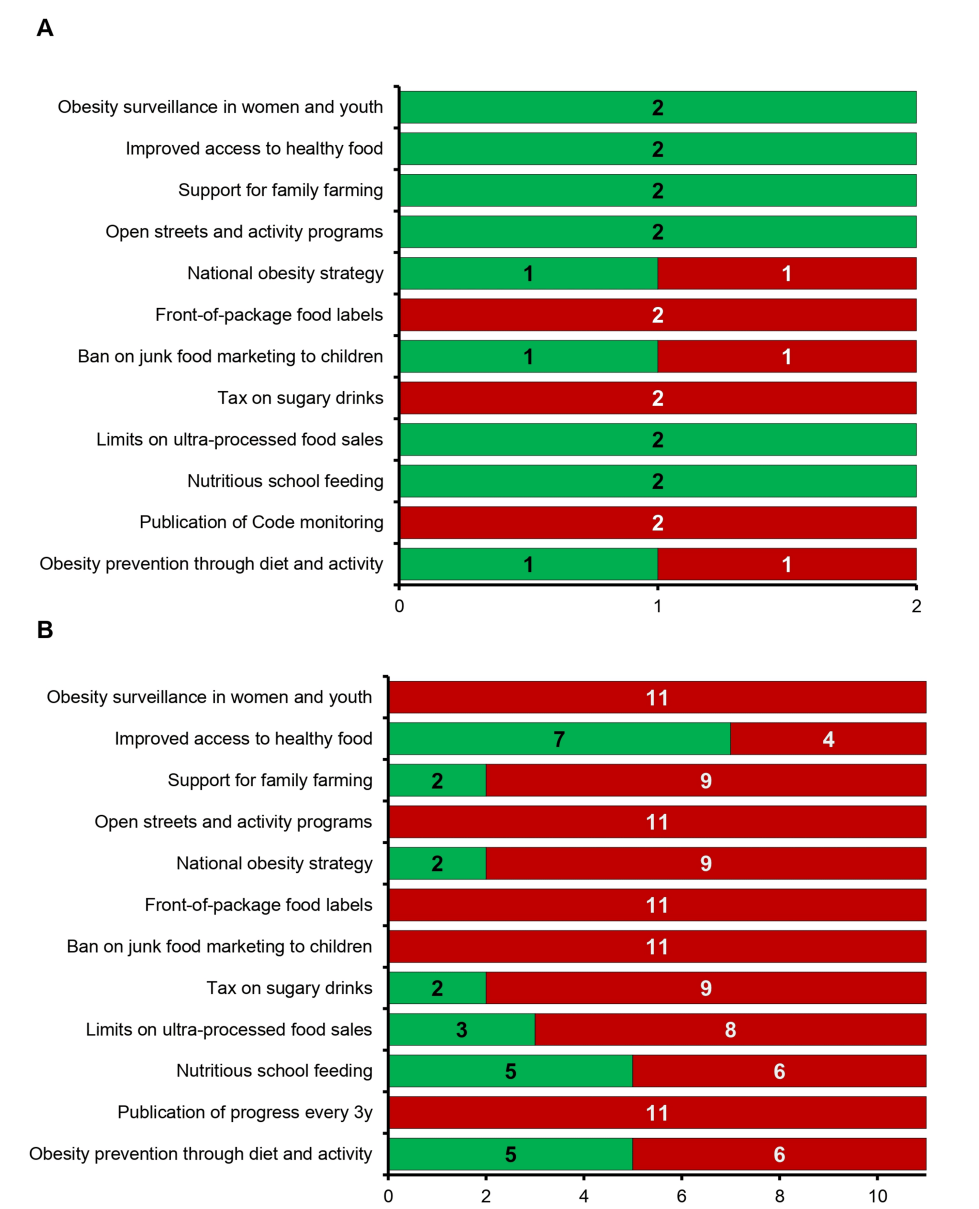
National progress on obesity policy implementation across Caribbean and North American countries (A) Proportion of policy actions completed across 12 indicators from PAHO’s Plan of Action, showing higher implementation in the USA and Canada (53%) compared to the non-Latin Caribbean (36%). (B) Specific areas of policy progress across countries, illustrating fragmented implementation in the Caribbean, with gaps in nutrition labeling, marketing restrictions, taxation, and obesity surveillance.

To assess how countries are responding to rising obesity and diabetes risk, we reviewed national progress on 12 policy measures outlined in PAHO’s Plan of Action for the Prevention of Obesity in Children and Adolescents (Figure 5). These indicators span areas such as fiscal policies, school nutrition, surveillance, and food environment regulation. In total, North American countries (USA and Canada) achieved 53% of indicators, while the non-Latin Caribbean implemented just 36%.

North America showed consistent policy development across sectors, with Canada and the United States meeting targets for obesity prevention in primary care, national school feeding, healthy food access, and dietary surveillance (Figure 5A). In contrast, policy action in the non-Latin Caribbean remained highly fragmented (Figure 5B). While several countries adopted programs supporting school feeding, open streets, and food access, key areas such as front-of-package labeling, junk food marketing bans, sugar-sweetened beverage taxation, and ongoing obesity surveillance were rarely achieved. Only two Caribbean nations reported any progress on nutrition labeling, while no countries had fully adopted front-of-package standards or regular publication of surveillance data.

The Bahamas met 6 of the 12 policy indicators, falling short in areas related to marketing restrictions, taxation, and labeling. These policy gaps may contribute to the persistently high rates of obesity and diabetes observed in the country. The limited progress across the region reflects broader structural barriers, including limited legislative capacity, fragmented health systems, and competing economic priorities. Without comprehensive implementation of evidence-based policies, efforts to reverse noncommunicable disease (NCD) trends may remain ineffective.

## Discussion

In this study, we examined diabetes and obesity trends in the Bahamas using international health and socioeconomic data. While the national prevalence of diabetes was 8.8%, nearly 29% of cases were estimated to be undiagnosed, and mortality under age 60 reached 6.2%, one of the highest rates observed. Women in The Bahamas had the highest obesity prevalence among all countries compared, along with elevated diabetes-related mortality. These findings indicate that prevalence alone does not capture the full extent of disease impact, particularly for women. By integrating data on detection, mortality, and gender disparities, this study offers a more comprehensive assessment of diabetes patterns in The Bahamas. These results are descriptive and observational, intended to highlight patterns rather than establish causal relationships.

While diabetes prevalence in The Bahamas appears moderate at 8.8%, this figure fails to reflect the extent of underdiagnosis and premature mortality. Mortality under age 60 reaches 6.2%, nearly twice that of the United States (3.5%) and among the highest in the Caribbean [23]. Nearly 29% of people with diabetes are undiagnosed, well above the regional estimate of 24.2% [12] and more than double the U.S. rate. These trends point to delayed diagnosis and inadequate care. Regional comparisons of prevalence and mortality reinforce this disconnect. Saint Kitts and Belize report much higher prevalence (16.1% and 14.5%, respectively) but only slightly higher mortality than The Bahamas. In contrast, Barbados and Antigua report lower mortality despite higher prevalence, likely reflecting more effective screening and management [11]. These outcomes are most pronounced among women in the Bahamas. Between 2012 and 2022, female obesity rose from 44.9% to 55.1%, and female diabetes mortality reached nearly 44 per 100,000-more than five times the U.S. rate of 7.9. This aligns with regional findings showing higher physical inactivity and obesity in women [24,25] and studies highlighting gendered barriers to diagnosis and disease management [7,10]. The national profile reflects not low prevalence but missed opportunities to detect and manage disease before complications arise, especially in women. While this analysis cannot confirm direct causal pathways, these patterns are consistent with systemic barriers in screening, access, and long-term care that warrant closer investigation.

Despite years of national and regional efforts, The Bahamas appears off track to meet global NCD targets, including SDG 3.4’s goal of reducing premature mortality by one-third by 2030. Persistently high under-60 mortality and undiagnosed diabetes suggest that strategies focused on behavior change alone are not producing the necessary impact. In the Bahamas, 8.8% of the population is living with diabetes, yet nearly 29% of cases remain undiagnosed, and mortality under age 60 has reached 6.2%, nearly double the rate reported in the United States [12]. These patterns point to systemic delays in diagnosis and gaps in long-term care, not just lifestyle risk. Regional comparisons reinforce this disconnect. Saint Kitts and Belize report far higher prevalence (16.1% and 14.5%), yet only slightly higher mortality than The Bahamas. In contrast, Barbados and Antigua report lower mortality despite higher prevalence differences that may reflect earlier detection or more consistent follow-up care [11]. These trends are most pronounced in women, where both obesity and mortality have risen sharply over the past decade. This aligns with regional literature showing that gender and socioeconomic factors influence how people interact with the health system and whether they receive timely care [10,24,25]. When viewed against global benchmarks, the Bahamian diabetes profile reflects a broader Caribbean pattern: national NCD strategies are often in place, but structural gaps persist, and outcomes remain unchanged. Even large-scale behavioral improvements proved insufficient to meet global targets in Jamaica, an outcome that underscores the need for systemic reform [8]. The Bahamas, like much of the region, faces the same constraint: not a lack of policy activity, but the limits of how progress has been defined and measured.

In the Bahamas, economic inequality, unstable employment, and food dependence shape who develops diabetes and who receives care. Imported food makes up 76% of the national supply [15], which limits the availability of affordable, nutritious options, especially for lower-income households. Food insecurity affects 17.2% of the population, and income inequality remains among the highest in the region, with a GINI coefficient of 0.57, both confirmed by validated national data sources [16]. These pressures are intensified by over-reliance on tourism, which has widened income gaps by concentrating earnings among the wealthy and leaving much of the workforce in low-wage, unstable service jobs [26,27]. For many Bahamians, these realities make it harder to maintain a healthy diet, seek regular care, or afford diabetes treatment over time.

Regional research supports this pattern. Limited financial protection, unstable employment, and under-resourced health systems continue to restrict access to early diagnosis and sustained diabetes care patterns well documented in Afro-Caribbean populations [10,28]. In the Bahamas, high out-of-pocket costs and inconsistent access to services contribute to late detection and poor management. Addressing these issues will require more than clinic-based interventions. Policies must account for the role that food access, wages, and household resources play in shaping health outcomes across the population.

The disconnect between national metrics and lived outcomes in The Bahamas suggests the need to revisit how progress in diabetes care is defined. Policies have largely targeted prevalence reduction, yet prevalence alone has remained stable even as underdiagnosis and premature mortality persist. The Bahamas currently meets only 6 of 12 PAHO policy indicators for obesity prevention, an outcome that reflects gaps in implementation, coordination, or both. While strategies such as school nutrition policies and SSB taxes have been introduced, their impact remains limited. In Barbados, for example, an SSB tax failed to produce meaningful dietary shifts due to aggressive marketing of untaxed alternatives and consumer confusion [29]. These challenges are not unique. In The Bahamas, where 76% of food is imported and public health messaging competes with a powerful commercial food environment, policies may be technically sound but functionally weak [15]. While these patterns suggest a disconnect between policy presence and health outcomes, this study’s descriptive, cross-sectional design cannot establish direct causal links between specific policy gaps and mortality outcomes. Instead, the findings highlight areas where closer evaluation of policy implementation and impact is needed. As a result, evaluation based on policy presence alone overstates progress. A more meaningful set of indicators would include rates of diagnosis, premature mortality, and implementation outcomes, not just the existence of plans.

Tools like FINDRISC offer promise for identifying undiagnosed cases, but their success depends on integrated systems that can link early screening to accessible care [30]. Without follow-up capacity, even effective detection strategies fail to change outcomes. Similar limitations affect behavioral campaigns and education efforts, which cannot achieve long-term impact without concurrent structural support. Metrics should capture what systems are actually delivering, not just what they intend to achieve, as shown in recent regional analyses [8]. For The Bahamas, that means a shift from tracking risk factors in isolation to evaluating the delivery and reach of health services, especially among women and low-income populations. Redefining success in these terms is essential if national efforts are to move beyond symbolic progress.

This analysis has several limitations. The reliance on cross-sectional, aggregate data precludes causal inference, limits assessment of changes over time, and does not capture dynamic interactions such as feedback loops, behavioral adaptation, or broader macroeconomic shifts. Estimates of underdiagnosis and premature mortality could not be consistently disaggregated by sex or socioeconomic status, restricting more granular subgroup analysis. Use of secondary, modeled estimates from international organizations (IDF, WHO, PAHO, FAO, CDB, IMF) ensured comparability but introduced uncertainty and potential reporting bias, as national systems vary in quality, completeness, and definitions. Missing data limited the inclusion of some indicators across countries, reducing regional consistency and comparability. These constraints limit precision and generalizability, but the study’s intent was to provide a comparative, systems-level perspective to highlight structural vulnerabilities rather than to generate individual-level predictions.

## Conclusions

This study highlights that diabetes and obesity in the Bahamas are not only widespread but also compounded by high underdiagnosis and premature mortality. Despite prevalence rates comparable to or lower than some high-income countries, outcomes in the Bahamas remain worse, reflecting persistent gaps in detection and care. Regional comparisons show the Bahamas faces some of the highest levels of obesity and food insecurity in the Caribbean, underscoring structural vulnerabilities that contribute to poor chronic disease outcomes.

Improving outcomes will require moving beyond policy adoption to implementation with measurable reach and impact. Strengthening primary care screening and treatment, addressing food environments and inequality, and enhancing surveillance systems are critical steps. Regional benchmarking can help identify feasible strategies, but national responses must be tailored to local conditions. Reframing success to include reductions in underdiagnosis, premature mortality, and inequities will be essential for developing more effective, equitable, and sustainable public health policies in The Bahamas and across the Caribbean.

## Appendices

Data availability

All data used in this study are publicly available and can be provided upon request. These sources include the World Health Organization (https://www.who.int/data), the International Diabetes Federation Diabetes Atlas (https://diabetesatlas.org/), the World Bank World Development Indicators (https://data.worldbank.org/indicator), the Pan American Health Organization Core Indicators Portal (https://opendata.paho.org/en/core-indicators), the Food and Agriculture Organization Food Security Statistics (https://www.fao.org/faostat/en/#data/FS), the WHO European Mortality Database (https://gateway.euro.who.int/en/datasets/european-mortality-database), the United Nations Development Programme Human Development Reports (https://hdr.undp.org/), the International Monetary Fund Selected Issues Reports (https://www.imf.org), and the Caribbean Development Bank Country Economic Reviews (https://www.caribank.org/publications-and-resources/resource-library/economic-reviews/country-economic-review-2022-dominica). 

**Table 1:** National indicators across Caribbean and comparator countries Diabetes prevalence and mortality data are from IDF (2021). Food import and insecurity estimates are based on FAO reports (2022 and 2019–2021 average). Human Development Index (HDI), Inequality-adjusted HDI (IHDI), and GINI coefficients were obtained from UNDP (2019 and 2022). Country codes follow ISO standards.

Source		IDF 2021	IDF 2021	FAO 2022	FAO 2019-2021	UNDP 2022	UNDP 2022	UNDP 2019	UNDP 2022
Country	Country code	Age-adjusted comparative prevalence of diabetes (%) 2021	Proportion of diabetes-related deaths in people under 60 years (%) 2021	Food Imports (% of Exports)	Food Insecurity (%)	HDI	IHDI	GINI	GINI
Antigua and Barbuda	ATG	11.70	4.80	489	33	0.826			
Bahamas	BHS	8.80	6.20	76	17.2	0.82	0.663	0.533	0.57
Barbados	BRB	14.00	3.30	84	31.1	0.809	0.617		
Belize	BLZ	14.50	10.60	38	42.3	0.7			
Dominica	DMA	11.70		270	34.4	0.74			
Grenada	GRD	12.60	4.50	328	22.3	0.793			
Guyana	GUY	11.70	6.90	10		0.742			
Saint Kitts and Nevis	KNA	16.10	9.90	85	26.9	0.838			
Saint Lucia	LCA	11.70	5.00	208		0.725	0.539		
Saint Vincent and the Grenadines	VCT	8.00	5.00	174	33.3	0.772			
Suriname	SUR	12.70	7.90	8	35.8	0.69			
USA	USA	10.70	3.50	9	8	0.927	0.823	0.415	0.413
France	FRA	5.30	1.50	9	5.9	0.91	0.82	0.312	0.307
Germany	DEU	6.90	1.70	5	3.5	0.95	0.881	0.318	0.317
Canada	CAN	7.70	2.00	2	9.3	0.94	0.865	0.317	0.29

**Table 2:** Age-standardized prevalence of obesity (BMI ≥ 30 kg/m²) among adults by sex and year (2012–2022) Data represent age-standardized estimates of obesity prevalence (%) among adults aged 18 and older in The Bahamas (BHS), United States (USA), France (FRA), and Germany (DEU) by sex from 2012 to 2022. Estimates were obtained from the WHO Global Health Observatory.

Country	Age Group En	Sex En	Year	Prevalence of obesity among adults, BMI ≥ 30 kg/m² (age-standardized estimate) (%)		
BHS	18+ years	Female	2012	44.94		
BHS	18+ years	Female	2013	45.98		
BHS	18+ years	Female	2014	47.02		
BHS	18+ years	Female	2015	48.06		
BHS	18+ years	Female	2016	49.10		
BHS	18+ years	Female	2017	50.12		
BHS	18+ years	Female	2018	51.13		
BHS	18+ years	Female	2019	52.13		
BHS	18+ years	Female	2020	53.11		
BHS	18+ years	Female	2021	54.09		
BHS	18+ years	Female	2022	55.06		
BHS	18+ years	Male	2012	34.19		
BHS	18+ years	Male	2013	34.67		
BHS	18+ years	Male	2014	35.13		
BHS	18+ years	Male	2015	35.57		
BHS	18+ years	Male	2016	36.01		
BHS	18+ years	Male	2017	36.45		
BHS	18+ years	Male	2018	36.88		
BHS	18+ years	Male	2019	37.32		
BHS	18+ years	Male	2020	37.78		
BHS	18+ years	Male	2021	38.25		
BHS	18+ years	Male	2022	38.73		
USA	18+ years	Female	2012	37.79		
USA	18+ years	Female	2013	38.35		
USA	18+ years	Female	2014	38.89		
USA	18+ years	Female	2015	39.44		
USA	18+ years	Female	2016	39.98		
USA	18+ years	Female	2017	40.52		
USA	18+ years	Female	2018	41.04		
USA	18+ years	Female	2019	41.57		
USA	18+ years	Female	2020	42.09		
USA	18+ years	Female	2021	42.62		
USA	18+ years	Female	2022	43.15		
USA	18+ years	Male	2012	36.07		
USA	18+ years	Male	2013	36.53		
USA	18+ years	Male	2014	36.99		
USA	18+ years	Male	2015	37.46		
USA	18+ years	Male	2016	37.94		
USA	18+ years	Male	2017	38.42		
USA	18+ years	Male	2018	38.90		
USA	18+ years	Male	2019	39.38		
USA	18+ years	Male	2020	39.86		
USA	18+ years	Male	2021	40.35		
USA	18+ years	Male	2022	40.85		
FRA	18+ years	Male	2012	11.74		
FRA	18+ years	Male	2013	11.53		
FRA	18+ years	Male	2014	11.32		
FRA	18+ years	Male	2015	11.11		
FRA	18+ years	Male	2016	10.91		
FRA	18+ years	Male	2017	10.71		
FRA	18+ years	Male	2018	10.52		
FRA	18+ years	Male	2019	10.33		
FRA	18+ years	Male	2020	10.16		
FRA	18+ years	Male	2021	9.99		
FRA	18+ years	Male	2022	9.82		
DEU	18+ years	Male	2012	21.97		
DEU	18+ years	Male	2013	21.96		
DEU	18+ years	Male	2014	21.95		
DEU	18+ years	Male	2015	21.96		
DEU	18+ years	Male	2016	21.99		
DEU	18+ years	Male	2017	22.03		
DEU	18+ years	Male	2018	22.07		
DEU	18+ years	Male	2019	22.12		
DEU	18+ years	Male	2020	22.18		
DEU	18+ years	Male	2021	22.25		
DEU	18+ years	Male	2022	22.33		
FRA	18+ years	Female	2012	11.76		
FRA	18+ years	Female	2013	11.56		
FRA	18+ years	Female	2014	11.35		
FRA	18+ years	Female	2015	11.13		
FRA	18+ years	Female	2016	10.91		
FRA	18+ years	Female	2017	10.68		
FRA	18+ years	Female	2018	10.46		
FRA	18+ years	Female	2019	10.24		
FRA	18+ years	Female	2020	10.02		
FRA	18+ years	Female	2021	9.82		
FRA	18+ years	Female	2022	9.62		
DEU	18+ years	Female	2012	18.85		
DEU	18+ years	Female	2013	18.82		
DEU	18+ years	Female	2014	18.79		
DEU	18+ years	Female	2015	18.75		
DEU	18+ years	Female	2016	18.71		
DEU	18+ years	Female	2017	18.66		
DEU	18+ years	Female	2018	18.61		
DEU	18+ years	Female	2019	18.55		
DEU	18+ years	Female	2020	18.5		
DEU	18+ years	Female	2021	18.46		
DEU	18+ years	Female	2022	18.43		

**Table 3:** Age-standardized mortality rates for diabetes mellitus (Excluding CKD Due to Diabetes) per 100,000 population in adults (≥18 Years) by country, sex, and year (2009–2019) Data show age-standardized mortality estimates for diabetes mellitus (excluding chronic kidney disease attributable to diabetes) from 2009 to 2019, stratified by country and sex. Rates are reported per 100,000 population for adults aged 18 and older, based on estimates from the Global Burden of Disease Study. Countries include The Bahamas (BHS), United States of America (USA), France (FRA), and Germany (DEU).

Location Name	Year	Sex	Diabetes mellitus (excluding CKD due to Diabetes) Age standardized per 100,000			
Bahamas	2009	Female	44.08			
Bahamas	2010	Female	42.65			
Bahamas	2011	Female	41.99			
Bahamas	2012	Female	39.69			
Bahamas	2013	Female	39.43			
Bahamas	2014	Female	37.60			
Bahamas	2015	Female	38.50			
Bahamas	2016	Female	38.05			
Bahamas	2017	Female	37.47			
Bahamas	2018	Female	38.94			
Bahamas	2019	Female	39.09			
United States of America	2009	Female	8.66			
United States of America	2010	Female	8.32			
United States of America	2011	Female	8.51			
United States of America	2012	Female	8.16			
United States of America	2013	Female	8.03			
United States of America	2014	Female	7.76			
United States of America	2015	Female	7.83			
United States of America	2016	Female	7.71			
United States of America	2017	Female	7.90			
United States of America	2018	Female	7.88			
United States of America	2019	Female	7.93			
Bahamas	2009	Male	41.42			
Bahamas	2010	Male	41.66			
Bahamas	2011	Male	41.30			
Bahamas	2012	Male	40.79			
Bahamas	2013	Male	37.62			
Bahamas	2014	Male	38.13			
Bahamas	2015	Male	39.67			
Bahamas	2016	Male	40.32			
Bahamas	2017	Male	39.66			
Bahamas	2018	Male	40.90			
Bahamas	2019	Male	41.35			
United States of America	2009	Male	13.22			
United States of America	2010	Male	12.76			
United States of America	2011	Male	13.16			
United States of America	2012	Male	12.72			
United States of America	2013	Male	12.63			
United States of America	2014	Male	12.63			
United States of America	2015	Male	12.82			
United States of America	2016	Male	12.84			
United States of America	2017	Male	13.39			
United States of America	2018	Male	13.36			
United States of America	2019	Male	13.59			
FRA	2009	Female	1.28			
FRA	2010	Female	1.18			
FRA	2011	Female	1.14			
FRA	2012	Female	1.21			
FRA	2013	Female	1.22			
FRA	2014	Female	1.13			
FRA	2015	Female	1.1			
FRA	2016	Female	1.07			
FRA	2017	Female	1.09			
FRA	2018	Female	0.91			
FRA	2019	Female	0.89			
DEU	2009	Female	1.52			
DEU	2010	Female	1.48			
DEU	2011	Female	1.63			
DEU	2012	Female	1.48			
DEU	2013	Female	1.51			
DEU	2014	Female	1.47			
DEU	2015	Female	1.51			
DEU	2016	Female	1.45			
DEU	2017	Female	1.51			
DEU	2018	Female	1.49			
DEU	2019	Female	1.39			
FRA	2009	Male	12.41			
FRA	2010	Male	11.72			
FRA	2011	Male	12.09			
FRA	2012	Male	11.97			
FRA	2013	Male	11.52			
FRA	2014	Male	11.04			
FRA	2015	Male	10.74			
FRA	2016	Male	10.45			
FRA	2017	Male	10.79			
FRA	2018	Male	9.97			
FRA	2019	Male	9.97			
DEU	2009	Male	15.46			
DEU	2010	Male	15.78			
DEU	2011	Male	15.71			
DEU	2012	Male	15.52			
DEU	2013	Male	16.33			
DEU	2014	Male	15.05			
DEU	2015	Male	15.95			
DEU	2016	Male	14.88			
DEU	2017	Male	15.9			
DEU	2018	Male	15.84			
DEU	2019	Male	15.1			

**Table 4:** National obesity-related policy indicators: achieved vs. not achieved status by country and policy domain This table presents the implementation status of national-level obesity prevention policies across countries, categorized by indicator code and description. Policies are grouped by domain, including school feeding, food labeling, marketing regulations, taxation, access to healthy food, and surveillance. Achievements are tallied per indicator, showing the number of countries that have implemented each measure (“Achieved”) versus those that have not (“Not Achieved”). Original indicator descriptions from the source dataset are included for reference.

Indicator Code	Indicator Description	Achieved	Not Achieved
1.1.1	Obesity prevention through diet and activity	5	6
2.2.1	Publication of progress every 3y		11
1.2.2	Nutritious school feeding	5	6
2.1.1	Limits on ultra-processed food sales	3	8
2.1.2	Tax on sugary drinks	2	9
3.1.1	Ban on junk food marketing to children		11
3.2.1	Front-of-package food labels		11
3.3.1	National obesity strategy	2	9
4.1.1	Open streets and activity programs		11
4.2.1	Support for family farming	2	9
4.3.1	Improved access to healthy food	7	4
4.3.2	Obesity surveillance in women and youth		11
5.1.1			
	Indicator Description	Achieved	Not Achieved
Indicator Code	Obesity prevention through diet and activity	1	1
1.1.1	Publication of Code monitoring		2
2.2.1	Nutritious school feeding	2	
1.2.2	Limits on ultra-processed food sales	2	
2.1.1	Tax on sugary drinks		2
2.1.2	Ban on junk food marketing to children	1	1
3.1.1	Front-of-package food labels		2
3.2.1	National obesity strategy	1	1
3.3.1	Open streets and activity programs	2	
4.1.1	Support for family farming	2	
4.2.1	Improved access to healthy food	2	
4.3.1	Obesity surveillance in women and youth	2	
4.3.2			
5.1.1			
	original names		
	Family obesity prevention through healthy eating and physical activity initiatives.		
	Publication of the results of Codeâ€™s monitoring process.		
	Countries in which maternity health services are certified as BFHI.		
	School feeding programmes that satisfy nutritional needs.		
	Norms that promote the sale of healthy foods and limit availability of ultra processed foods.		
	Tax on sugar-sweetened beverages and other calorie high products.		
	Regulations to protect children and adolescents from marketing of unhealthy foods.		
	Norms regarding front-of-package labelling in ultra processed and sugar sweetened beverages.		
	Multisectoral implementation of a plan of action to tackle obesity.		
	Open streets and physical activity.		
	Family farming programs.		
	Measures to better relative prices and access to healthy foods.		
	Overweight and obesity prevalence surveillance in pregnant women, adolescents, and children.		
